# Kikuchi-Fujimoto Disease from Eastern India

**DOI:** 10.4103/0974-777X.68539

**Published:** 2010

**Authors:** Pankaj Singhania, Rudrajit Paul, S Maitra, AK Banerjee, MA Hashmi

**Affiliations:** 1 *Department of Medicine, Medical College and Hospitals, Kolkata, India*; 2 *Eko CT and MRI Scan Center, Medical College and Hospitals Campus, Kolkata, India*

**Keywords:** Arthritis, Histiocytic necrotising lymphadenitis, Systemic lupus erythematosus (SLE)

## Abstract

Kikuchi’s disease, a rare disorder which usually presents with fever painful lymphadenopathy, rash and arthritis, all of which are close mimickers of infective and immunological disorders. It is essentially a histopathological diagnosis and tests to rule out other connective tissue disorders or infective etiology must be undertaken. We present a series of two cases of kikuchi-fujimoto’s disease presenting primarily with lymphadenopathy and fever in all cases. The first is a case of generalized lymphadenopathy and the second case of kikuchi’s disease with SLE, a rare association. Lymph node excision biopsy and histopathology documented Kikuchi Fujimoto disease in above cases. All the cases improved on follow up and had no residual stigmata.

## CASE REPORT

A 32-year-old man presented with tender, discrete, soft, mobile lymph nodes in both axillae and right cervical region of one-month duration, with occasional fever. The lymph node histopathology showed pictures of Kikuchi-Fujimoto disease. At follow-up his lymph nodes had regressed significantly on symptomatic medicines.

**Figure 1:**
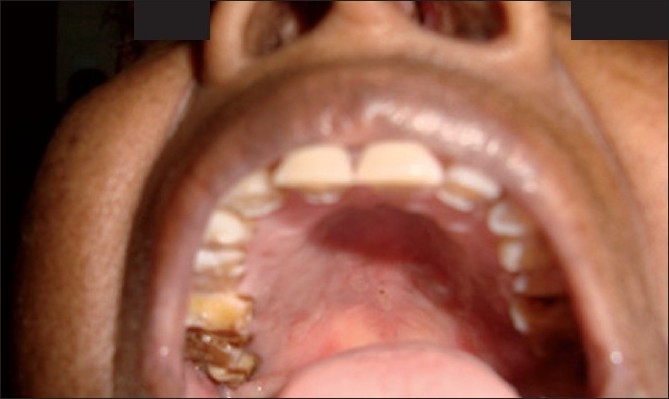
Oral ulcer in case 2

**Figure 2:**
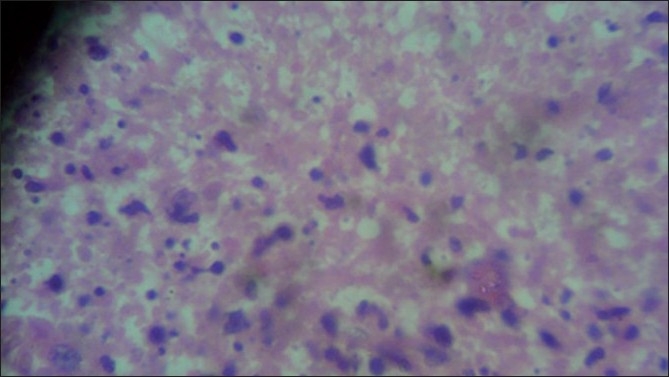
Typical lymph node biopsy showing Histiocytes and macrophages

Second case was that of a 55-year-old housewife who presented with high fever, polyarthritis, anasarca and oral ulcers [Figure 1]. There was nontender mobile lymphadenopathy in cervical, axillary and inguinal regions. USG abdomen revealed enlarged echogenic kidneys. ANF was positive (1:640; homogeneous); anti dsDNA= neg; 24-hour urinary protein was 7 g. Kidney biopsy showed features of lupus nephritis, WHO stage 2. A biopsy from axillary lymph node showed Kikuchi’s disease [Figure 2]. The patient was given oral steroids with anti-rheumatic drugs for SLE. By three months, her lymph nodes regressed.

## DISCUSSION

**Figure 3:**
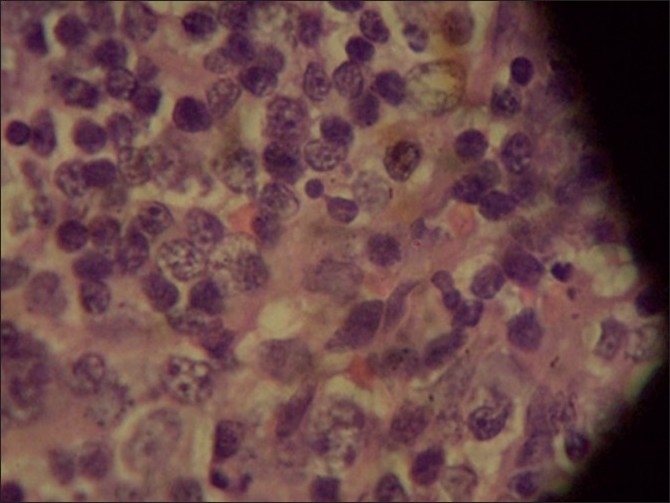
Lymphnode histopathology show paracortical necrosis, Histiocytes(crescentic nuclei) & Karyorrhexis

Kikuchi’s disease, histiocytic necrotising lymphadenitis, is a rare self-limiting disorder with fever, painful lymphadenopathy, rash and arthritis.[1] It is a histopathological diagnosis, and other infective and immunological disorders must be ruled out.[2] Both viral and immunologic etiologies are postulated.[3] The cardinal features are fever, painful cervical lymphadenopathy, rash or arthritis. The diagnosis is made by lymph node histopathology, which shows: para-cortical necrosis, histiocytes (crescentic nuclei) and karyorrhexis [Figures 1 and 3].[4]

There are few reports of overlap of Kikuchi’s disease, adult Stills disease, SLE and arthritis.[5–8] Whether SLE has any causative role is not known.[9–12] However, a distinct entity of necrotizing lymphadenitis in SLE mimicking Kikuchi’s disease but responding to steroids and immunomodulators has been proposed.[13,14] Kikuchi’s disease is usually reported in young population and rarely over 50 years of age,[10] like our case.

## CONCLUSION AND TEACHING POINTS

Kikuchi’s disease can present like infective lymphadenopathy and should be one of the differential diagnoses of enlarged lymph nodes. Histological examination is very important to distinguish it and avoid unnecessary treatment. Lymph nodes regress completely on symptomatic medicines without any residual stigma.
